# Exploring the Antiangiogenic and Anti-Inflammatory Potential of Homoisoflavonoids: Target Identification Using Biotin Probes

**DOI:** 10.3390/biom14070785

**Published:** 2024-06-30

**Authors:** Xiang Fei, Sangil Kwon, Jinyoung Jang, Minyoung Seo, Seongwon Yu, Timothy W. Corson, Seung-Yong Seo

**Affiliations:** 1 College of Pharmacy, Gachon University, Incheon 21936, Republic of Korea; drfei@gachon.ac.kr (X.F.); tkddlf26@gachon.ac.kr (S.K.); dkck12@gachon.ac.kr (J.J.); netmin32@naver.com (M.S.); racer9679@gachon.ac.kr (S.Y.); 2 Leslie Dan Faculty of Pharmacy, University of Toronto, Toronto, ON M5S 3M2, Canada

**Keywords:** homoisoflavonoid, photoaffinity labeling, target identification, natural products

## Abstract

Chemical proteomics using biotin probes of natural products have significantly advanced our understanding of molecular targets and therapeutic potential. This review highlights recent progress in the application of biotin probes of homoisoflavonoids for identifying binding proteins and elucidating mechanisms of action. Notably, homoisoflavonoids exhibit antiangiogenic, anti-inflammatory, and antidiabetic effects. A combination of biotin probes, pull-down assays, mass spectrometry, and molecular modeling has revealed how natural products and their derivatives interact with several proteins such as ferrochelatase (FECH), soluble epoxide hydrolase (sEH), inosine monophosphate dehydrogenase 2 (IMPDH2), phosphodiesterase 4 (PDE4), and deoxyhypusine hydroxylase (DOHH). These target identification approaches pave the way for new therapeutic avenues, especially in the fields of oncology and ophthalmology. Future research aimed at expanding the repertoire of target identification using biotin probes of homoisoflavonoids promises to further elucidate the complex mechanisms and develop new drug candidates.

## 1. Introduction

**Figure 1:**
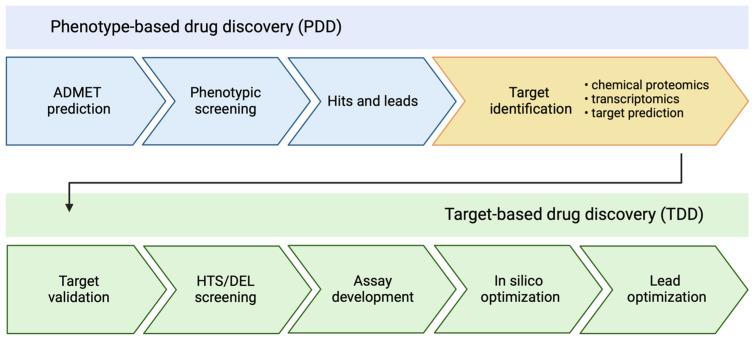
Chemical proteomics to link phenotype-based drug discovery and target-based drug discovery.

Chemical proteomics has emerged as a crucial technique for the identification and validation of target proteins in medicinal chemistry and chemical biology. Examples of fluorescence-based biosensors include the Green Fluorescent Protein (GFP) used for monitoring gene expression and protein localization, the FRET (Förster Resonance Energy Transfer) biosensors for detecting protein-protein interactions, and calcium indicators like Fluo-4 for measuring intracellular calcium levels. Additionally, label-free protein target identification methods for natural active products further exemplify the integration of these technologies [1,2,3]. The major challenge in phenotypic screening approaches is the difficult and complex process of identifying cellular targets and deciphering the modes of action (MOA) of identified hits [4]. Despite these challenges, identifying target proteins in phenotype-based drug discovery presents several advantages, including clarification of the MOA and easier transition to target-based process. This transition allows the use of structure-activity relationships (SAR) to steer medicinal chemistry efforts towards refining lead compounds. Consequently, successful target identification becomes a critical, yet rate-limiting step in some drug discovery processes [5]. Identifying the proteins that interact with a small molecule is a critical task in various chemical biology contexts [6]. Figure 1 shows two principal strategies in drug discovery: phenotype-based and target-based drug discovery (PDD and TDD, respectively) [7]. Target identification using biotin probes serves as a versatile tool bridging PDD and TDD. Recent trends in PDD initiate with in silico ADMET prediction techniques to forecast potential therapeutic compounds based on computational models and simulations. These methods leverage machine learning, cheminformatics, and molecular modeling tools to virtually screen large chemical libraries and identify promising candidates [8]. This initial in silico screening reduces the number of compounds for subsequent phenotypic screening assays, accelerating the hit and lead identification stages. For instance, ADMETlab 3.0 offers comprehensive online ADMET prediction, increasing screening with broader coverage, improved performance, API functionality, and decision support, which are crucial for early-stage drug discovery [9]. After the in silico prediction phase, the workflow progresses to phenotypic screening, where compounds are evaluated in cell-based or organism-based assays to assess their effects on specific phenotypes or disease models. The promising hits from these assays are further optimized through iterative cycles of chemical modifications and phenotypic testing to improve their potency, selectivity, and pharmacokinetic properties, leading to the selection of lead compounds. Target identification techniques, such as chemical proteomics, transcriptomics, and computational target prediction methods, are then employed to elucidate the mechanisms of action and identify the molecular targets of these lead compounds [10]. This step provides insights into the biological pathways modulated by the compounds and guides further optimization efforts.

Conversely, in the TDD approach, the process commences with target validation, where the relevance and viability of a biological target (e.g., a protein or enzyme) for a particular disease or therapeutic area are established through various experimental and computational methods. Once a promising target is identified, high-throughput screening (HTS) or DNA-encoded library (DEL) screening techniques are employed to identify compounds that modulate the target’s activity. In silico prediction methods, such as structure-based virtual screening, ligand-based modeling, and machine learning approaches, are often used to prioritize and enrich the chemical libraries for these screening campaigns, improving the hit rates and reducing the experimental workload. The next step is to develop assays that can reliably and sensitively detect the interactions between the target and the possible drug candidates. The identified hits are then improved and fine-tuned using optimization techniques, such as molecular docking, QSAR modeling, and molecular dynamics simulations, which enhance their potency, selectivity, and pharmacokinetic properties. In lead optimization the most promising compounds are further optimized through iterative cycles of chemical modifications, guided by in silico predictions and experimental validation, to develop potential drug candidates suitable for preclinical and clinical studies.

Target identification has helped reveal the mechanisms of biologically active natural products in medicinal chemistry research [11]. Moreover, finding the targets of hit compounds that produce a desired phenotype is crucial in cell-based and other phenotypic small molecule screens for discovering drugs or probes [12]. It is also essential to verify that target-directed compounds bind the expected proteins and to detect any off-targets [13]. This confirmation step is crucial for establishing the specificity of chemical probes such as biotin probes [14]. Biotin-tagged probes are a common approach for identifying the protein targets of bioactive natural products using chemical proteomics. The general strategy involves attaching a biotin tag to the natural product of interest through a chemical linker. The probe with a biotin tag is added to a cell lysate or living cells to enable it to attach to target proteins. The complexes of probe and protein are then separated using streptavidin beads that bind very strongly to biotin. The isolated proteins are released and detected by mass spectrometry [15].

**Figure 2:**
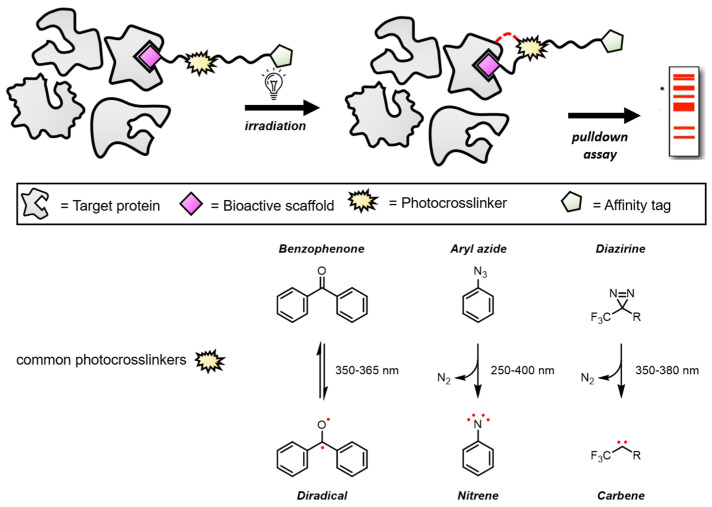
Photoaffinity labeling for chemical proteomics. The asterisk (*) indicates the bands on the gel where the target protein is located. The red dots represent electrons generated by the photocrosslinker upon UV activation. These electrons form intermediates that readily bond covalently with cysteine residues near the binding pockets of the target protein and ligand, enhancing the compound’s interaction with the target protein.

Biologically active natural products engage with target proteins predominantly through non-covalent interactions such as hydrogen bonds, ionic bonds, and hydrophobic forces. These interactions are crucial for the bioactivity of these molecules. However, the non-covalent nature of these bonds can sometimes lead to disadvantages, such as suboptimal activity results, when attempting to elucidate the precise mechanisms of action or identify specific protein targets. Photoaffinity labeling (PAL) is an advanced type of biotin probe and has emerged as a powerful tool to address these challenges. PAL probes based on small molecules react irreversibly with a target protein when activated by UV irradiation, as shown in Figure 2, allowing for the identification of binding interactions within the complex cellular environment. This facilitates the target identification, thereby leading to the phenotype-based drug discovery process.

The general design of photoaffinity probes involves the incorporation of an affinity unit, a photoreactive moiety, and an identification/reporter tag, such as biotin. The photoreactive moiety allows for the photo-inducible covalent attachment to targets, and the biotin tag is vital for the detection and isolation of probe–protein adducts. Photocrosslinkers that form covalent bonds to target proteins have emerged as essential tools for PAL. Despite their importance, there are only three powerful photocrosslinkers currently available: benzophenone, aryl azide, and diazirine. Their molecular structures and the reactive intermediates are illustrated in Figure 2. When exposed to light, each of these photocrosslinkers generates a reactive intermediate that can then form covalent bonds, typically carbon–carbon bonds, with nearby amino acid residues on the target protein.

**Figure 3:**
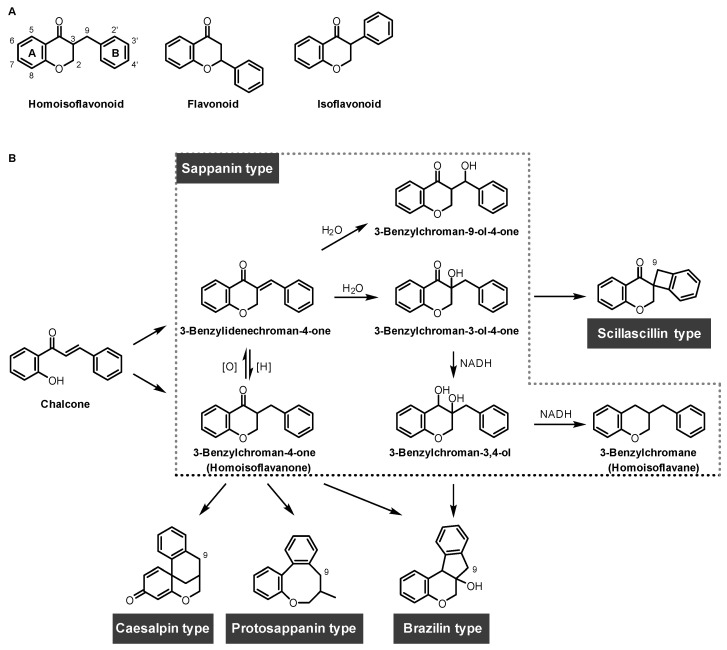
(**A**) Comparative structures of homoisoflavonoid scaffold, flavonoid, and isoflavonoid; (**B**) biosynthetic pathway and structures of five types of homoisoflavonoids.

Homoisoflavonoids are a special type of flavonoid, which are uncommon in nature but have diverse sources in different plant families (Figure 3). Over 300 homoisoflavonoids were reported in *Asparageace*, *Fabaceae*, *Portulacaceae*, *Cucurbitaceae*, and *Polygonaceae* families [16,17]. Homoisoflavonoids have 16 carbon atoms and feature two aromatic rings and a heterocyclic ring containing oxygen atoms [18,19]. The main structural difference between homoisoflavonoids and conventional isoflavonoids is that homoisoflavonoids have an extra alkyl carbon (C9 in Figure 3A) that isoflavonoids lack. Their chemical structure allows for a unique balance of rigidity and flexibility, enabling them to form diverse interactions with protein targets through hydrophobic interactions, π-π, π-cation interactions, and hydrogen bonds. Homoisoflavonoids are distinctive 16-carbon compounds that come from chalcone through different biosynthetic pathways as suggested by the Lopez group. These pathways generate different types, such as sappanin, scillascillin, brazilin, caesalpin, and protosappanin, which are shown in Figure 3B [20].

**Figure 4:**
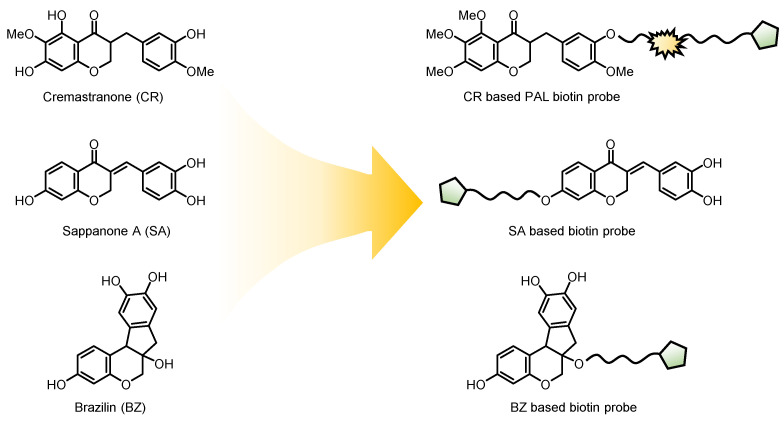
Design of biotin probes based on homoisoflavonoids.

The unique structural features and diverse sources of homoisoflavonoids distinguish them from traditional isoflavonoids, showcasing their potential for therapeutic applications in various diseases [21,22]. One of the most promising therapeutic applications of homoisoflavonoids such as cremastranone (CR) is their antiangiogenic activity, particularly in treating ocular diseases [23,24]. Similarly, sappanone A (SA) and brazilin (BZ) have shown significant anti-inflammatory and antioxidant activities [25]. However, the proteins that interact with homoisoflavonoids are mostly unidentified. As Figure 4 illustrates, researchers employ methods such as biotin probes, photoaffinity labeling, and chemical proteomics to uncover the target protein.

## 2. Applications of Homoisoflavonoid Probes in Target Identification

In this section, we will review some notable cases where target proteins in different diseases and biological processes have been successfully identified by homoisoflavonoid probes. This covers target identification of ferrochelatase (FECH), soluble epoxide hydrolase (sEH), inosine monophosphate dehydrogenase 2 (IMPDH2), phosphodiesterase 4 (PDE4), and deoxyhypusine hydroxylase (DOHH) by various probes (Table 1).

### 2.1. Cremastranone Inhibited Ocular Neovascularization by Targeting FECH

**Figure 5:**
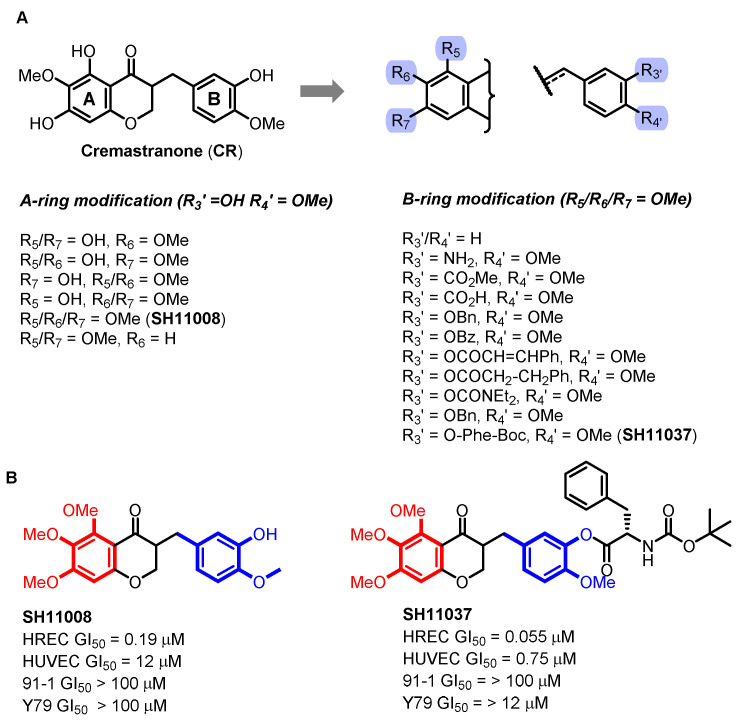
Structure of cremastranone and its derivatives. (**A**) Cremastranone and design of homoisoflavonoid derivatives; (**B**) structure of antiangiogenic SH11008 and SH11037.

*Cremastra appendiculata* (Orchidaceae) is a medicinal plant in East Asia, particularly valued for its tumor metastasis prevention properties. The pseudobulbs of *C. appendiculata* are the source of cremastranone (CR), a compound identified for its potent antiangiogenic properties, initially isolated by H. J. Kwon and colleagues [26]. This compound sparked significant interest due to its potential as a treatment for eye diseases characterized by choroidal and retinal neovascularization [27,28]. These therapeutic findings prompted the first attempts at its total synthesis by the Seo group, aiming to explore the antiangiogenic capabilities of homoisoflavanones like CR [29]. As Figure 5A shows, a series of substituents were strategically introduced to the A- and B-rings of the CR derivatives. Among these derivatives, SH11008, characterized by its trimethoxy groups at the R5, R6, and R7 positions of the A-ring, demonstrated a selectivity profile favoring human retinal endothelial cells (HRECs) over other cell types, such as human umbilical vein endothelial cells (HUVECs), as illustrated in Figure 5B. This finding highlighted the potential for targeted therapy in retinal conditions without affecting other endothelial cells significantly. To optimize the potency, further modifications were explored at the R3′ position of SH11008, leading to the synthesis of aryl esters and amide analogs incorporating various *N*-protected amino acids. One notable derivative, SH11037, with Boc-Phe-OH coupled to SH11008, exhibited markedly enhanced activity in HRECs, evidenced by a GI_50_ value of 55 nM. The selectivity of SH11037 was profound, inhibiting the proliferation of HRECs 14-fold more effectively than HUVECs, 218-fold more than Y79 retinoblastoma cells, and over 1000-fold more than 92-1 uveal melanoma cell.

**Figure 6:**
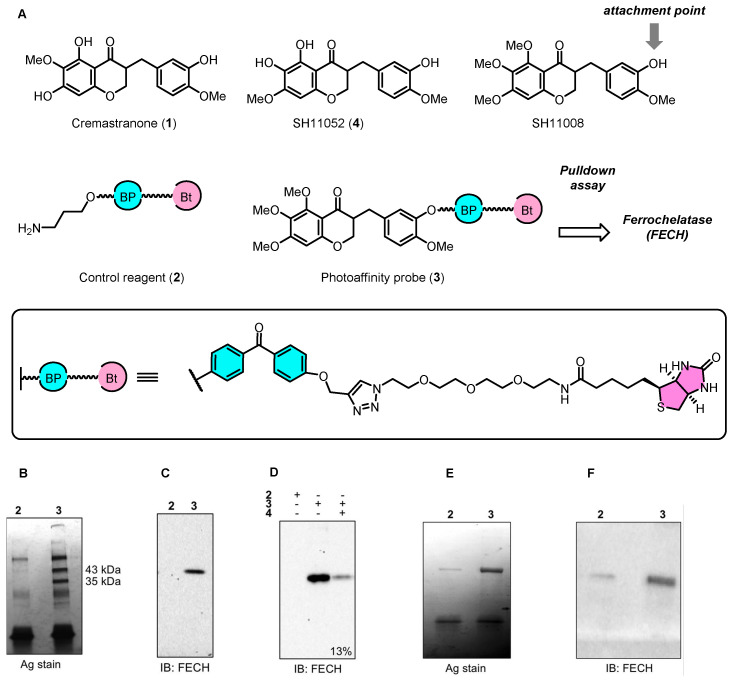
Identification of FECH as a target of the antiangiogenic natural product, cremastranone. (**A**) Chemical structures of cremastranone (**1**), control reagent (**2**), and photoaffinity probe (**3**). (**B**) Silver staining of SDS-PAGE-separated proteins from photoaffinity chromatography with indicated reagents showed specific bands at 43 and 35 kDa, which were excised and proteomically identified. The upper band was FECH. (**C**) Immunoblot of proteins that were isolated using an antibody against FECH. (**D**) Immunoblot of proteins extracted from a competition assay with excess active cremastranone isomer SH11052 (**4**). (**E**) Silver-stained SDS-PAGE gel of recombinant human FECH protein pulled down using photoaffinity chromatography. (**F**) Anti-FECH immunoblot of a similar pul-down experiment. Figure adapted from Figure 1 in Ref. [30].

Using chemical proteomics methods, researchers have made considerable progress in finding out which proteins are targeted and how CR and its derivatives block blood vessel formation. As shown in Figure 6, developing photoaffinity probes for SH11008 helped to discover FECH as a target protein [30]. Initially, affinity reagents based on SH11008 were used in a photo-affinity pulldown assay to identify protein interactions. This assay revealed distinct FECH protein bands in the treated samples, but not in the control samples, indicating that the compound binds specifically to FECH (Figure 6B). To confirm the identity of the pulled-down protein, an immunoblot using an antibody against FECH was performed. The presence of a clear band in lane 3 (photoaffinity probe-treated sample) confirms that FECH is one of the target proteins (Figure 6C). This was further validated by competitive binding assays (Figure 6D). This assay tests whether an active isomer of cremastranone (SH11052) can compete with the photoaffinity probe for binding to FECH. The significant reduction of the FECH signal in the presence of SH11052 (+, +) indicates effective competition, further validating FECH as a target. Silver staining of recombinant human FECH pulled down using the photoaffinity probe confirms that the probe can isolate FECH from a mixture, consolidating its identification as a target protein (Figure 6E). Finally, similar to Figure 6C, the immunoblot shown in Figure 6E confirms the presence of FECH in the proteins pulled down by the photoaffinity probe, reinforcing the evidence that FECH is a target of cremastranone.

Progressing with the development of small-molecule inhibitors targeting ferrochelatase (FECH) opens a significant pathway for broadening treatment options for neovascular eye disease patients resistant to current anti-VEGF therapies, addressing a vital medical need. The pinpointing of FECH as a crucial target in combating such diseases has propelled research into identifying FECH inhibitors as promising therapeutic candidates. A high-throughput screening strategy was pivotal in uncovering potent inhibitors of FECH activity. The high-throughput screening (HTS) campaign focusing on triazolopyrimidinone derivatives has led to significant progress in the development of drug-like inhibitors of ferrochelatase (FECH). Among the compounds identified, SH17023 emerged as a notable candidate, characterized by its triazolopyrimidinone scaffold [31]. The acquisition of the X-ray co-crystal of FECH and the ligand enhanced the understanding of their interactive mechanisms.

**Figure 7:**
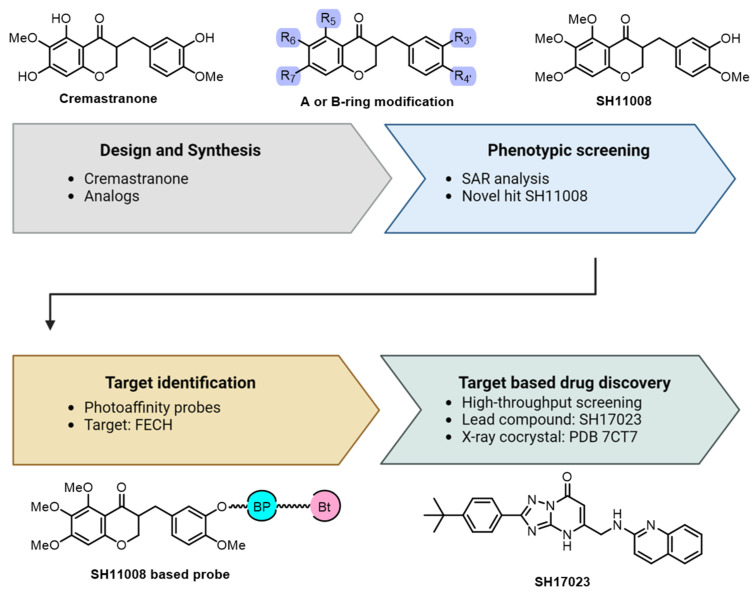
Exploring the molecular target of cremastranone and developing FECH inhibitors.

Figure 7 demonstrates that the procedure started with the total synthesis of cremastranone, and was followed by producing derivatives of homoisoflavonoid. In an effort to enhance the effectiveness and specificity of cremastranone as a suppressant of proliferation in human retinal microvascular endothelial cells (HRECs), a range of homoisoflavonoid derivatives were synthesized and their biological activities assessed. For instance, modifications were made at the C3’ position on the B-ring by adding various N-substituted amino acids, which significantly increased the compound’s ability to inhibit HREC proliferation. Among the synthesized derivatives, phenylalanyl-incorporated analog SH11037 demonstrated the most potent activity and remarkable selectivity, which was confirmed through dose-dependent inhibition assays of HREC proliferation, migration, and tube formation. Concurrently, a photoaffinity probe was employed for target identification. Once the target was established, X-ray co-crystallization elucidated the molecular interactions. They aimed to improve cremastranone’s ability and specificity as a blocker of the growth of retinal blood vessel cells (HREC). They changed the C3 position of the B-ring, adding various N-substituted amino acids, which greatly enhanced the blocking effect on HREC growth. Among the created derivatives, the one with phenylalanine added, SH11037, showed the strongest effect and remarkable selectivity, which was verified by testing how it reduced HREC growth, movement, and tube formation at different doses, and aided in the design of new scaffold target inhibitors. The culmination of this process is the target-based drug discovery, aiming to develop therapeutics for specific diseases by inhibiting the function of the identified target proteins. This specific research direction not only shows the power of these compounds in blocking FECH activity but also introduces a new way to combat ocular blood vessel growth and possibly other conditions related to FECH activity.

### 2.2. Chemical Proteomics Identified sEH as a Therapeutic Target for Ocular Neovascularization

**Figure 8:**
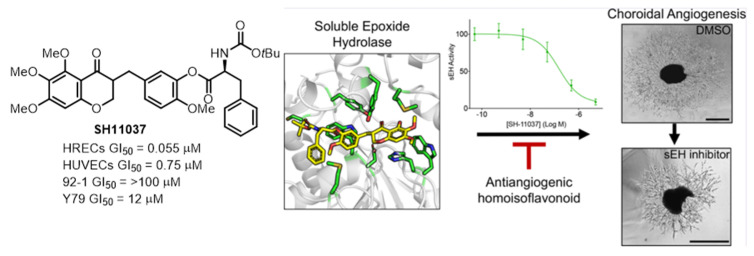
Homoisoflavonoid analog SH11037 inhibition of sEH demonstrates its potential as an anti-angiogenic therapeutic agent. Figure adapted from Figure 1 in Ref. [34].

SH11037, a synthetic homoisoflavonoid derived from cremastranone, demonstrated strong antiangiogenic effects against choroidal neovascularization (CNV) without causing ocular toxicity [32,33]. As shown in Figure 8, SH11037 has shown synergistic effects when combined with anti-VEGF therapy in reducing L-CNV [34]. These findings suggest that SH11037 holds promise as a therapeutic approach to manage ocular neovascularization, particularly when used in conjunction with existing treatments.

**Figure 9:**
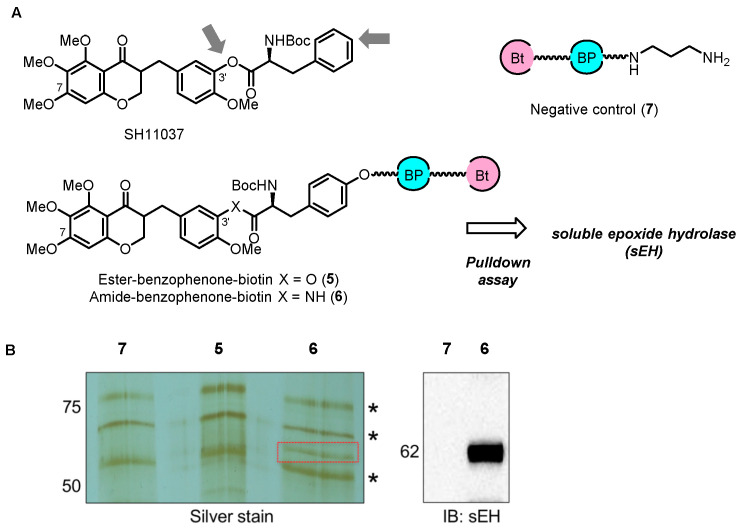
(**A**) Structures of SH11037-based photoaffinity probes. (**B**) Proteins isolated with indicated reagents were resolved by SDS-PAGE and silver stained, then identified by mass spectrometry. A distinct band was present in pull-down with **6** but not **5** or **7** (red box); asterisks represent nonspecific bands. MW in kDa indicated. Figure adapted from Figure 1 in Ref. [35].

In order to determine if the target(s) of SH11037 differed from those of cremastranone, the Seo group first synthesized two photoaffinity reagents, **5** and **6** [35]. The ester group in **5** was replaced by an amide in **6** for increased stability (Figure 9). Additionally, a control compound **7** lacking the homoisoflavanone moiety was included. The phenolic OH group at the C3’ position was initially considered for linking the photoaffinity reagent due to its importance in improving the antiangiogenic effect when substituted with the *N*-Boc-phenylalanyl moiety. Despite the strategic design, introducing a tether to the NHBoc group site in SH11037 presented several challenges, including low reactivity of the amino group and instability of the ester group during Boc-deprotection or when linking with a carbamate. These photoaffinity probes, which were designed and synthesized despite some synthetic difficulties, are an important advance in finding and characterizing the molecular targets of homoisoflavonoids. Affinity reagent **6** isolated a specific protein target, which was identified as sEH, and immunoblotting also verified the identity of the isolated protein [36].

Epoxy-fatty acids (EpFAs), such as epoxyeicosatrienoic acids (EETs), epoxydocosapentaenoic acids (EpDPEs), and epoxyeicosatetraenoic acids (EpETEs), have been recognized as natural signaling molecules with anti-inflammatory properties [36]. sEH is a key enzyme that catalyzes the hydrolysis of these EpFAs, thereby playing a critical role in modulating inflammation and angiogenesis. Emerging evidence has highlighted sEH as a potential therapeutic target for ocular neovascularization [37], making SH11037 an intriguing novel scaffold for targeting this enzyme.

### 2.3. Sappanone A Inhibited IMPDH for Anti-Inflamatory Effects

**Figure 10:**
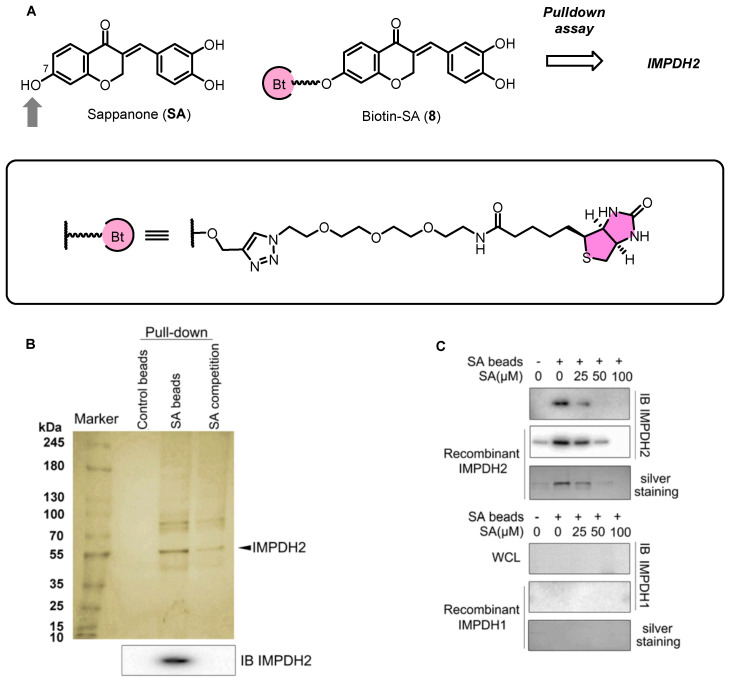
(**A**) Structure of SA and its biotin probe. (**B**) Identification of SA target proteins using pull-down technology coupled with shotgun proteomics. (**C**) SA selectively binds to IMPDH2. Figure adapted from Figure 1 in Ref. [38].

Sappanone A (SA) is a homoisoflavonoid derived from *Biancaea sappan* that shows promising anti-inflammatory and antioxidant properties. Researchers discovered that SA effectively inhibits microglial activation, as evidenced by a significant reduction in the release of inflammatory markers and gene expressions in BV-2 cells and primary microglia. To identify the pharmacological target of SA, they prepared chemical probes for affinity purification and fluorescent labeling [38]. As shown in Figure 10, a biotin-tagged SA probe (Biotin-SA, **8**) was used to pull down the cellular target of SA. The probe **8** retained the ability to inhibit NO release, suggesting that the chemical modification did not influence the biological activity of SA. Pull-down assay coupled with stable isotope labeling with amino acids in cell culture (SILAC) revealed IMPDH2 as a potential key target. Subsequent pull-down assays coupled with shotgun proteomics further confirmed IMPDH2 as the primary target of SA. The interaction between SA and IMPDH2 was verified through several experiments. IMPDH2 was pulled down by SA beads, which as detected by Western blotting and silver staining. Moreover, an excess amount of SA effectively blocked the binding of IMPDH2 to SA beads. Meanwhile, the authors did not detect obvious binding of SA with the IMPDH1. This is particularly interesting since over the past decade IMPDH has been identified as a key target for the development of immunosuppressive and anti-inflammatory drugs. However, the lack of selectivity among current IMPDH inhibitors limits their clinical application due to adverse effects. In further studies, SA was shown to increase the stability of IMPDH2, suggesting a ligand–protein complex formation. From cellular thermal shift assay (CETSA), they found that SA treatment efficiently protected IMPDH2 protein from temperature-dependent degradation. Then, DARTS assay also demonstrated a concentration-dependent reduced proteolysis with the incubation of SA.

By selectively inhibiting IMPDH2, SA significantly reduced inflammatory markers and gene expression in microglial activation, without the undesired binding to IMPDH1. This specificity stems from SA’s covalent bonding to the cysteine thiols on IMPDH2, particularly at the Cys140 residue, which does not occur with IMPDH1 due to its structural differences. Molecular dynamics simulations revealed that SA binds within the IMPDH2 Bateman domain, altering its conformation and thereby inhibiting its function. This interaction leads to decreased tetramer formation of IMPDH2, impacting downstream inflammatory signaling pathways like NF-κB and p38 MAPK. Notably, SA’s administration has shown significant neuroprotective and anti-inflammatory effects in vivo without notable hematological side effects, highlighting its potential for high safety and efficacy.

Additionally, beyond IMPDH2 inhibition, SA has also been identified as a potent phosphodiesterase 4 (PDE4) inhibitor [39]. This dual functionality is crucial for addressing diseases like chronic obstructive pulmonary disease (COPD), as inflammation and oxidative stress play pivotal roles in these conditions. Through molecular docking, literature investigation, and pharmacophore analysis, SA was recognized for significant anti-inflammatory and antioxidant activities. Further study focused on designing and synthesizing a novel class of PDE4 inhibitors with antioxidant properties, aimed at treating COPD.

### 2.4. Brazilin Allosteric Activate DOHH for Neuroprotection in Ischemic Stroke

Brazilin (BZ) is a natural bioactive small molecule derived from *Caesalpinia sappan*, a medicinal plant traditionally used for the treatment of ischemic stroke. This bioactive phytochemical has garnered significant attention in recent years due to its diverse pharmacological activities and therapeutic potential. Extensive medicinal chemistry research has been conducted on brazilin, including studies on its total synthesis [8,9,10], SAR, and biological mechanisms of action [40]. Researchers have explored brazilin and its derivatives as potential therapeutic agents for various diseases, such as hypercholesterolemia [41], inflammatory conditions [42], and neurodegenerative conditions like Parkinson’s disease [43].

Deoxyhypusine hydroxylase (DOHH) is an enzyme essential for the hypusine modification of eIF5A, a unique cellular protein. It plays a vital role in cellular functions such as proliferation, differentiation, and apoptosis, and is implicated in diseases like cancer, malaria, and HIV-1. The DOHH/eIF5A pathway, crucial for nerve regeneration, underscores DOHH’s potential as a therapeutic target for ischemic neuronal injury, despite the lack of selective small molecule DOHH activators for stroke therapy to date.

**Figure 11:**
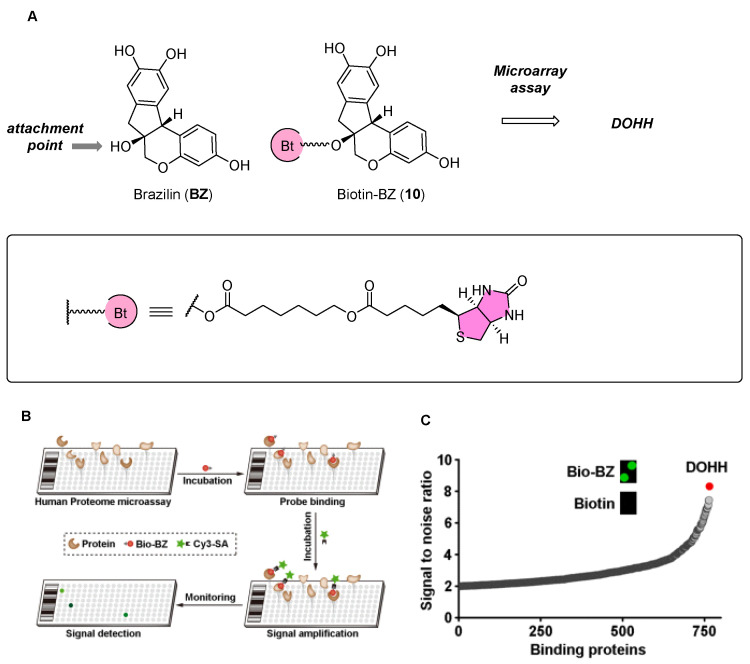
(**A**) Structure of BZ and its biotin probe **10**. (**B**) Identification of BZ target proteins using human proteome microarray. (**C**) DOHH was identified as the protein with the highest signal-to-noise ratio (SNR) when screening for BZ-binding proteins using a human proteome microarray. Figure adapted from Figure 2 in Ref. [44].

Utilizing a biotin probe **9** and the HuProt™ human proteome microarray, researchers have identified DOHH as a target of BZ (Figure 11). The interaction between BZ and DOHH is marked by an allosteric induction of conformational changes within DOHH, notably at the Cys232 residue. This interaction boosts DOHH’s catalytic activity, which subsequently promotes mitophagy, a crucial process for neuroprotection both in laboratory settings and living organisms. The study’s insights elevate DOHH to the status of a promising pharmacological target for treating ischemic neuronal injury. Furthermore, it heralds BZ as the first-known allosteric activator of DOHH, laying the groundwork for a novel category of therapeutic agents aimed at combating human ischemic stroke. This innovation opens new avenues for drug design, with the potential to transform the therapeutic approach to ischemic stroke [44].

### 2.5. Emerging Techniques for Target Identification beyond PAL

While PAL remains a valuable tool, a broader range of advanced techniques have emerged for comprehensive target identification, including protein microarrays for high-throughput screening of protein interactions, DARTS (drug affinity responsive target stability) that leverages drug-induced protein stabilization, and the integration of big data analytics with high-throughput screening to predict potential targets and elucidate biological networks. Emerging trends further expand these capabilities, with chemical proteomic techniques like ABPP (activity-based protein profiling) and TPP (thermal proteome profiling) enabling proteome-wide studies of small molecule–protein interactions and mechanisms of action. Genome-wide CRISPR-Cas9 knockout screens identify genetic vulnerabilities and targets essential for cell survival upon drug treatment. Moreover, advances in single-cell proteomics provide unprecedented insights into heterogeneous target populations within tissues and granular cellular responses to drugs. These multifaceted approaches, complementing traditional PAL methods, are driving target identification efforts and facilitating the development of novel therapeutics targeting critical disease pathways [45].

## 3. Conclusions

In conclusion, this review has summarized key developments in the identification of the targets of homoisoflavonoids, highlighting their possible applications for novel treatments. Cremastranone’s interaction with FECH presents a novel pathway in treating ocular neovascular diseases, broadening the scope beyond traditional anti-VEGF therapies. The involvement of sEH in the action of SH11037 suggests a promising strategy for augmenting current treatments for wet AMD. Furthermore, the selective inhibition of IMPDH2 by sappanone A suggests a targeted approach to neuroinflammatory conditions, potentially minimizing side effects. Additionally, identifying sappanone A as a PDE4 inhibitor highlights the potential of natural compounds to develop dual-action treatments for COPD, targeting both inflammation and oxidative stress. Moreover, the identification of deoxyhypusine hydroxylase (DOHH) as a novel pharmacological target for ischemic stroke, with the natural compound brazilin showing strong binding affinity and neuroprotective effects, opens new paths for drug design aimed at enhancing DOHH activity to reduce ischemic neuronal injury. These findings emphasize the integration of chemical proteomics in drug discovery, highlighting the ongoing importance of natural products as a source for new therapeutic agents. The insights derived from these approaches could steer future research towards more precise and efficient drug development models.

## Figures and Tables

**Table 1:** Target identification of homoisoflavonoids using various chemical proteomics methods.

Probe Structure	Methods	Target	Disease
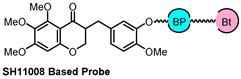	PAL and X-ray co-crystal	FECH	angiogenesis
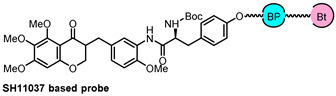	PAL and MD simulation	sEH	angiogenesis
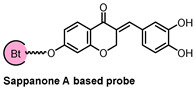	CETSA, DARTS, and MD simulation	IMPDH2	inflammatory
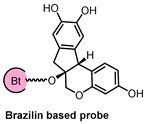	Microarray, CETSA, Molecular Docking Simulation	DOHH	Ischemic stroke
